# Does school ethos explain the relationship between value-added education and teenage substance use? A cohort study

**DOI:** 10.1016/j.socscimed.2012.02.045

**Published:** 2012-07

**Authors:** Wolfgang A. Markham, Robert Young, Helen Sweeting, Patrick West, Paul Aveyard

**Affiliations:** a School of Health and Social Studies, University of Warwick, Coventry CV4 7AL, UK; b MRC Social & Public Health Sciences Unit, 4 Lilybank Gardens, Glasgow G12 8RZ, UK; c UK Centre for Tobacco Control Studies, Primary Care Clinical Sciences, University of Birmingham, Birmingham B15 2TT, UK

**Keywords:** Teenagers, Substance use, School ethos, Value-added education, Scotland, Adolescents

## Abstract

Previous studies found lower substance use in schools achieving better examination and truancy results than expected, given their pupil populations (high value-added schools). This study examines whether these findings are replicated in West Scotland and whether school ethos indicators focussing on pupils' perceptions of schooling (environment, involvement, engagement and teacher–pupil relations) mediate the associations. Teenagers from forty-one schools (S2, aged 13, *n* = 2268; S4, aged 15, *n* = 2096) previously surveyed in primary school (aged 11, *n* = 2482) were surveyed in the late 1990s. School value-added scores were derived from standardised residuals of two regression equations separately predicting from pupils' socio-demographic characteristics (1) proportions of pupils passing five Scottish Standard Grade Examinations, and (2) half-day truancy loss. Outcomes were current smoking, monthly drinking, ever illicit drug use. Random effects logistic regression models adjusted for potential pupil-level confounders were used to assess (1) associations between substance use and school-level value-added scores and (2) whether these associations were mediated by pupils' perceptions of schooling or other school-level factors (school roll, religious denomination and mean aggregated school-level ethos scores). Against expectations, value-added education was positively associated with smoking (Odds Ratios [95% confidence intervals] for one standard deviation increase in value-added scores were 1.28 [1.02–1.61] in S2 and 1.13 [1.00–1.27] in S4) and positively but weakly and non-significantly associated with drinking and drug use. Engagement and positive teacher–pupil relations were strongly and negatively associated with all substance use outcomes at both ages. Other school-level factors appeared weakly and largely non-significantly related to substance use. Value-added scores were unrelated to school ethos measures and no ethos measure mediated associations between value-added education and substance use. We conclude that substance use in Scotland is more likely in high value-added schools, among disengaged students and those with poorer student–teacher relationships. Understanding the underpinning mechanisms is a potentially important public health concern.

## Highlights

► Contrary to previous studies, high value-added schools were associated with greater substance use prevalence in West Scotland. ► High value-added schools occurred throughout the socio-economic spectrum and ranges of exam results and truancy rates. ► Substance use in Scotland is more likely among disengaged students and those with poorer student–teacher relationships. ► Associations between value-added education and substance use were not mediated by pupils' perceptions of school ethos. ► Scottish schools may influence pupils' substance use through at least two pathways related to school ethos.

## Introduction

The prevalence of tobacco, alcohol and illicit drug use (substance use) varies markedly between schools. Although it is often assumed that such variation is explained by the social composition of pupils, this assumption is contradicted by the evidence. While teenage substance use varies according to characteristics such as gender or parental behaviours, these factors are unlikely to be able to account for between-school substance use variation (Aveyard, Markham, & Cheng, 2004a; Aveyard et al., 2005; West, Sweeting, & Leyland, 2004). To paraphrase Geoffrey Rose, the causes of variation in substance use between populations differ from the causes of substance use within populations (Rose, 1985).

Between-school substance use variations have been investigated from several different perspectives. One approach, based on what is termed school ethos or culture, suggests that the way schools operate in general, unrelated to what they do regarding health education lessons and interventions, is an important contextual risk factor for substance use. This model was examined by both Aveyard and Markham (Aveyard et al., 2004b; Markham et al., 2008) and West and colleagues (West et al., 2004). However, Aveyard and Markham (Aveyard et al., 2004b; Markham et al., 2008) and West et al. (2004) drew on different theoretical frameworks and used different methods to assess and measure school ethos.

Markham and Aveyard (2003) drew primarily on Bernstein's theory of cultural transmission (Bernstein, 1977) but also on theoretical conceptions of parenting (Baumrind, 1971) in proposing that school ethos might be conceived in terms of support and control practices. Thus, schools providing support for learning and behavioural controls appropriate for pupils' cultural expectations should achieve higher examination success and lower truancy rates than expected, given pupils' socio-demographic characteristics (higher value-added schools). These schools should also be more successful at encouraging pupils to adopt school values that are anti-substance use. In a test of these ideas, Markham and Aveyard defined high value-added schools as those with better than predicted exam results and lower than predicted truancy rates, when predictions are based on the socio-demographic characteristics of schools' pupil populations. In three English studies and one American study, tobacco, alcohol, and illicit drug use rates were, as predicted, lower in high value-added schools (Aveyard et al., 2004b; Bisset, Markham, & Aveyard, 2007; Markham et al., 2008; Tobler, Komro, Dabroski, Aveyard, & Markham, 2011). In the West Midlands, England, value-added education explained about 14% of the inter-school variation in smoking prevalence in years 7 and 9 (ages 11–14), and around 5% in year 11 (ages 15–16) (Aveyard et al., 2004b).^5^

West et al. (2004) drew on theoretical ideas from the school effects and health promoting schools fields. They proposed that pupils in more effective schools would have fewer health-risk behaviours (e.g. smoking) than might be expected given known predictors such as socio-demographic characteristics. These effective schools engage their pupils and foster good pupil–teacher relationships (MacBeath, Thomson, Arrowsmith, & Forbes, 1992; Mortimer, Sammons, Stoll, Lewis, & Ecob, 1988; Rutter, Maughan, Mortimore, & Ouston, 1979). Data from a Scottish cohort study, which included pupils' perceptions of the school environment, involvement with school, engagement with school and relationships with teachers, were used to examine the relationships between indicators of school ethos and teenage substance use. Substance use was higher in schools with higher levels of pupil self-reported disengagement, where pupils knew fewer teachers and, although less consistently, in larger schools.

The current investigation aimed to bring the two approaches together, using West and colleagues' Scottish dataset (West et al., 2004). This combined approach is valuable because value-added education as applied by Markham and Aveyard is measured very indirectly (see Methods), while West's and colleagues' perceptions of school ethos were obtained directly from pupils. We investigated three hypotheses:

1. School-level value-added education is associated with pupils' perceptions of school ethos (perceptions of school environment, school involvement, school engagement, teacher–pupil relationships).
2. School-level value-added education is negatively associated with school-level substance (smoking, alcohol consumption, and illicit drug) use, replicating Aveyard and Markham's previous findings.
3. Associations between school-level value-added education and substance use are mediated by pupils' perceptions of the school and/or by other school-level factors.

## Methods

### Sample

We used data from the ‘*West of Scotland 11-16 Study*’ a cohort of Scottish pupils resident in Glasgow and surrounding areas (West et al., 2004). Mainstream secondary schools (*n* = 41; two private schools were excluded for comparability with Markham and Aveyard's earlier studies) were randomly selected within strata based on geographical location, religious status (Catholic/non-denominational) and socio-economic status. Associated local authority primary schools (feeder schools and schools making large numbers of placing requests; *n* = 133) were sampled on the basis of the proportion of pupils transferring to the selected secondary schools.

Pupils recruited at the end of primary school (P7; aged 11) were followed-up two and four years later at S2 (aged 13; late 1996) and S4 (aged 15; early 1999). Sample sizes were 2482 at baseline, 2268 (91.4% of baseline) at S2 and 2096 (84.4% of baseline) at S4.

Confidential self-completion questionnaires were administered in school under exam conditions. The ‘11-16 Study’ received approval from all participating local authorities, schools and head teachers prior to each sweep of data collection. Ethical permission from the University of Glasgow Interim Ethics Committee for Non-Clinical Research Involving Human Subjects was received for the age 13 (S2 – in 1996) and 15 (S4 – in 1999) sweeps.

### Measures

#### Health behaviour outcomes

The outcomes, described in detail elsewhere (West et al., 2004), were current smoking (occasional/regular), monthly or more frequent drinking, and ever use of illicit drugs (including cannabis, magic mushrooms, temazepam, amphetamine, LSD, ecstasy, solvents, cocaine and heroin).

#### Value-added education

We used nationally available data (which schools are legally required to collect) on public examination results and truancy rates, for the five year period 1993–1997. We included data from all mainstream secondary schools in Glasgow and surrounding areas (*n* = 115) to improve precision. For each school, we calculated the mean proportions of: a) S4 pupils who passed five or more Scottish Standard Grade Examinations with grades between 1 and 4 (SG1-4); and b) half days lost to truancy for the whole school. Two logistic regression models were created using SG1-4 and truancy proportions as outcomes and two indicators of each school's socio-demographic profile as predictors: first, the proportion of pupils entitled to a clothing grant, indicating parent/guardian receipt of State financial support; second, area-based deprivation, defined as the mean deprivation score, based on address, for the main feeder primary schools (commonly 3–5 schools) to each selected secondary school. We assumed most primary school children attend local primary schools in the neighbourhoods where they live (Granville, Laird, Barber, & Rait, 2002).

The standardised residuals from these two logistic regression models represent the difference between the observed and expected SG1-4 examination passes and observed and expected truancy rates when expectations are based upon each school's socio-demographic profile.

Principal components analysis of the standardised residuals identified a single factor that explained 75.4% of the variance and had factor loadings of 0.87 for both SG1-4 and truancy residuals. This continuous variable constitutes the value-added score and reflects both adjusted examination success and adjusted truancy rates. Schools with a value-added score of zero have observed examination results and truancy rates equal to those expected. Scores of +1 and −1 respectively represent schools with a one standard deviation (1SD) above- and below-average performance.

#### Potential confounders

Factors other than attributes of secondary schools might predict teenage substance use. Those included in the study are described in detail elsewhere (West et al., 2004). Briefly, they include data on potential confounders that were collected at baseline (P7), namely, use of cigarettes or alcohol, or having been offered drugs in primary school, baseline (primary school) engagement, area deprivation derived from pupils' home postcodes (Carstairs, 1991) and pupils' religion (Protestant, Roman Catholic, other, none, and missing), Data on other potential confounders were collected at both S2 and S4 including age, gender, accurate and reliable parental or pupil reports of head of household social class (West, Sweeting, & Speed, 2001), personal income, family composition (both birth parents, step- and lone-parent families), parental care and control (Brief Parental Bonding Instrument; Klimidis, Minas, & Ata, 1992) and parental smoking and alcohol consumption.

#### Pupils' perceptions of school (PP)

Pupils' perceptions of school, informed by MacBeath et al.'s school ethos measures (MacBeath et al., 1992), described in detail elsewhere (West et al., 2004) comprised: school environment (physical and teacher-related aspects e.g. playground; teacher control); school involvement (e.g. feeling part of school, able to share worries with teachers); school engagement (e.g. thinking school a waste of time, skipping school); and teacher–pupil relationships (single question focussing on how many teachers pupils get on well with). Each measure was standardised. Higher scores represented more negative perceptions.

#### School-level factors (SLF)

School-level factors were school roll (number of pupils), religious denomination (Catholic or non-denominational), and aggregated school-level ethos (each school's mean score across the four dimensions of pupils' perceptions of school).

#### Analyses

We performed all modelling using Multilevel Modelling for Windows (MLwiN) with second-order penalised quasi-likelihood methods and random effects logistic regression models.

#### Associations between value-added education and pupils' perceptions of school

Hypothesis 1 was that school-level value-added education is associated with pupils' perceptions of school ethos. We included pupils' perceptions at both S2 and S4 (environment, involvement, engagement and teacher–pupil relationships) as outcomes in multilevel models, with value-added score as a predictor and adjustment for potential confounders (age, gender, social class, deprivation, religion, family structure, parental care and parental control, personal income, prior school engagement with primary school).

#### The association between school culture and substance use

Hypothesis 2 was that school-level value-added education is negatively associated with school-level substance use and Hypothesis 3 was that these associations are mediated by pupils' perceptions of school ethos and/or by other school-level factors. We created six random effects logistic regression models for each substance use outcome at both S2 and S4. To examine change in school-level variance in successive models, cases with missing data were excluded, reducing the sample by up to 12%.

1. Null models had substance use as the outcome with no predictors and quantified the school-level variance of each substance use outcome.
2. Individual Adjusted models examined school-level substance use variance after adjustment for potential confounders (outlined above; age, gender, social class, deprivation, religion, family structure, parental care and parental control, personal income, prior school engagement with primary school). Additionally, parental smoking or drinking were respectively included when current smoking or monthly drinking was the outcome and both parental smoking and drinking were included when ever use of illicit drugs was the outcome.
3. Model VA variants (Individual Adjusted models plus value-added education) examined the additional effect on school-level variance of adjusting for value-added score and also the strength of associations between value-added score and each substance use outcome. Odds Ratios [95% confidence intervals] for 1SD increase in value-added scores were calculated.
4. Model VA + PP variants examined whether pupils' perceptions of school (environment, involvement, engagement, teacher–pupil relationships) were associated with substance use and mediated any associations between value-added score and substance use.
5. Model VA + SLF variants examined whether school-level factors (school roll, denomination and aggregated school-level ethos) were associated with substance use and mediated any associations between value-added score and substance use.
6. Full model variants entered all confounders, value-added score, pupils' perceptions and school-level factors.

## Results

Schools providing both above- and below-average value-added education occurred throughout the socio-economic spectrum and the ranges of examination results and truancy rates (results available on request).

The prevalence of current smoking, monthly drinking and ever use of illicit drugs varied markedly between schools at both S2 and S4 (Table 1). Adding confounders potentially associated with risk of smoking, drinking, and experience of drugs (Individually Adjusted models) did not reduce the unexplained variation between schools (school-level variance) of current smoking and illicit drug use at S2 (Table 1). However, these confounders explained some between-school variation in monthly drinking at both S2 and S4, together with most between-school variation in illicit drug use and all between-school variation in current smoking at S4.

### Associations between value-added education and pupils' perceptions of school

Value-added scores had very weak and mostly non-significant associations with most pupils' perceptions (Table 2). The only (near) significant relationships were between higher value-added scores and better environment ratings (S2 only) and greater involvement with school (S2 and S4).

### Associations between value-added education, school ethos and substance use

Adding value-added scores to the Individual Adjusted models (Model VA) occasionally reduced the school-level variance of substance use but any reductions were modest (Table 3). High value-added education was associated with a modest increase in substance use at both S2 and S4, except for drinking at S4. However, only the associations with smoking at both S2 and S4 were statistically significant.

Adding pupils' perceptions of school (Model VA + PP) further reduced the school-level variance in smoking at S2 and drinking at both ages; the variance in drug use at S2 and smoking at S4 remained the same, while that of drug use at S4 increased slightly (Table 3). The associations between value-added education and substance use were not greatly changed by adding these potential mediators. Disengaged pupils and those who got on with fewer teachers were more likely to use all substances at both S2 and S4. Drinking at S2 was also significantly higher among pupils providing worse school environment ratings.

The addition of school-level factors (Model VA + SLF) reduced school-level substance use variance, almost eliminating that in S2 smoking (Table 4). The strength of the associations between value-added education and substance use were largely unchanged after adjustment for the school-level factors. Pupils attending larger schools were more likely to smoke at S2 and those attending schools with poorer overall ethos were more likely to smoke at both S2 and S4 and use illicit drugs at S2. School denomination had no statistically significant association with any substance use outcome.

In the Full Models (Table 5), the unexplained school-level variance in smoking at S2 was almost completely attenuated, while that in drinking at both ages and drugs at S2 was halved. The significant associations between the substance use outcomes and value-added education, pupils' perceptions of school and most school-level factors largely remained in the fully adjusted models. However, associations between overall school ethos and substance use were markedly attenuated, suggesting possible mediation.

## Discussion

In contrast to four previous studies, this analysis of Scottish teenagers found positive associations between value-added education and substance use. Thus, at both S2 and S4, current smoking was more likely in schools with better than expected examination results and truancy rates, given pupils' socio-demographic characteristics. These high value-added schools were also positively but weakly and largely non-significantly associated with early onset of alcohol and illicit drug use. Value-added education was largely unrelated to pupils' perceptions of school and adjusting for these perceptions did not change associations between value-added scores and substance use. The findings suggest that in the context of this study, value-added education and pupils' perceptions of school ethos have distinctly different associations with at least some forms of substance use. Additionally, schools with poorer overall pupil-rated ethos had higher levels of current smoking at S2 and S4 and illicit drug use at S2. However, the models suggest this could be because these schools had more pupils who individually rated the ethos poorly and it was these particular pupils who were at greater risk of substance use onset. Pupils providing good ethos ratings were no more at risk of substance use in poor ethos schools than in other schools, but the relatively small number of included schools and measurement error regarding aggregated school ethos preclude strong conclusions.

### Strengths and limitations

Three potential study limitations related to chance, bias or confounding may have affected the validity of our observed associations. In relation to chance, we hypothesised that value-added education influences smoking, drinking and illicit drug use, and our dataset allowed us to test this at two different ages for three outcomes, thus generating six statistical tests. It is therefore possible that the unexpected associations between value-added education and substance use are chance findings, but the consistent pattern suggests otherwise.

In relation to bias, the sampling of schools was random, so selection bias is an unlikely explanation of the results. Loss to follow-up occurred, but it was low and it seems unlikely that differential loss to follow-up in each school would have generated spurious associations between value-added education or school ethos indicators and substance use. This view is supported by subsequent analyses using weighted data. Weights to adjust for attrition within this sample were calculated and applied for each wave. The results using weighted data were substantively no different from those using unweighted data, accordingly we report only unweighted results.

Unmeasured and/or uncontrolled confounding is a major concern of all cohort studies. The dataset used here included relatively comprehensive data on potential confounders including prior (primary school) substance use behaviours, engagement with primary school, disposable income, family structure and parental bonding. Controlling for these potential confounders, which are commonly absent in other school effects and substance use studies, greatly reduces the possible influence of residual confounding. Having said this, while we have been careful to adjust for many key influences on teenage substance use, it is possible that we have omitted an influential, but unmeasured variable that may alter our findings.

In addition, the Scottish and English education systems are different. Hence, the method of measuring value-added education differed slightly from previously used methods in three ways. First, we calculated the proportion of pupils achieving at least 5 Scottish Standard Grades at 1–4, equivalent to English grades A–D, not grades A–C as used previously. Second, for three of the five years, the annual proportion of half days lost to truancy was rounded down to the nearest whole figure, which is important because truancy rates are low (typically 0.5%). Third, we included fewer measures of pupils' socio-demographic characteristics than we had previously done. These differences will have led to non-differential misclassification of schools achieving better than expected exam results and truancy rates and undermined our ability to select high value-added schools. However, this imprecision is unlikely to account for reverse findings to those expected.

### The nature of the influence of value-added education on substance use

Four previous studies in England and the US (Aveyard et al., 2004b; Bisset et al., 2007; Markham et al., 2008; Tobler et al., 2011) used analogous methods and found high value-added schools had lower risk of pupil substance use. We concluded this association was probably causal, mainly because findings across studies were consistent and alternative explanations were lacking. The contrary findings of this study cast doubt on our previous conclusion. Why might value-added education be related to increased substance use, and why might this occur in Scotland but not elsewhere?

With respect to the first question, ‘Why might value-added education be related to increased substance use?’, high value-added education may not, as hypothesised, be related to good quality control and support and may instead be related to repression (Aveyard et al., 2004b). Pupils' reaction to this repression may vary. One reaction might be rebellion manifested as substance use while another reaction in a different context might be submissive acquiescence manifested as abstinence from substance use.

Alternatively, high value-added education may commonly be related to good quality control and support as hypothesised. However, this relationship may depend on sampling schools whose primary educational goals are examination success and good attendance, thereby demonstrating the importance of context. Schools providing good quality support and control may not always focus solely on examination success and truancy, but may in addition focus on broader educational outcomes (e.g. alternative curricula, performance in sports or arts) and be relatively tolerant of truancy. Such schools would thus not necessarily achieve high value-added education scores but may be associated with decreased substance use. In contrast, schools providing poorer quality control and support in the same context may focus primarily on traditional academic examination results and truancy rates and could have both higher value-added scores and higher substance use prevalence.

Another possible explanation is that in some contexts, a minority of pupils may feel alienated or detached from the provision of good quality control and support and may consequently be disengaged and have poor teacher–pupil relations. This minority may not greatly influence the positive examination and truancy results of all students. However, if detached pupils tend to be heavy substance users, and excessive substance use is relatively uncommon, this minority may strongly influence school-level indicators of substance use. Fletcher, Bonell, and Rhodes (2009) drew on qualitative data to report that detached students attending high achieving English secondary schools are at much greater risk of substance use for a variety of reasons. The reasons included mood control, coping and bonding. Henderson, Ecob, Wight, and Abraham (2008) found that poor teacher–pupil relationships had a stronger influence on smoking uptake among boys attending affluent schools than among boys attending non-affluent schools in Scotland. They reasoned that affluent schools were more likely to have an academic focus than non-affluent schools and the effects of poor–teacher relationships on substance use would be amplified within the context of a strong academic focus (Henderson et al., 2008). Some of the high value-added schools in the study reported here would have had relatively low unadjusted academic attainment scores and relatively high unadjusted truancy rates. Hence, it is unlikely these high value-added schools would be commonly considered as high achieving or academic. Thus, the proposals of Fletcher et al. (2009) and Henderson et al. (2008) that particular sub-groups of detached pupils are at greater risk of substance use in respectively high achieving schools or schools with an academic focus may potentially be extended. Within a Scottish context, detached pupils attending schools that have relatively good examination results and truancy rates given the socio-demographic profile of their pupil populations, may be at increased risk of substance use.

A second question is why might high value-added education be related to increased substance use in the West of Scotland or Scotland more generally but not elsewhere? Glasgow populations have poorer mortality outcomes than populations with equal health determinants in the two most comparable UK cities (Liverpool, Manchester) (Walsh, Bendel, Jones, & Hanlon, 2010). The so called ‘Glasgow effect’ may possibly extend beyond mortality/morbidity outcomes and influence the relationships between value-added education and substance use examined in this study. Another potential explanation, given the continuing importance of religion for several areas of social life, including education, in the West of Scotland (Abbotts, Sweeting, Williams, & West, 2001), is that Catholic schools within the study had an uncommonly strong influence on pupil development. The influence of value-added education may as a consequence vary according to school denomination. However, we found no evidence that interactions between value-added education and denomination significantly influenced any substance use outcome in any model at either S2 or S4 (results available on request).

Factors associated with being educated in Scotland generally may also potentially alter the relationships between value-added education and substance use. When the data were collected, the Health Promoting School (HPS) initiative was widely embraced in Scotland but less so in England (Denman, Moon, Parsons, & Stears, 2002; West, 2006). The implementation of this initiative may have promoted pupil engagement and good teacher–pupil relations and, additionally, had a greater influence on substance use than the provision of high value-added education. High value-added schools in Scotland may have opted to focus on traditional educational outcomes rather than implement the HPS initiative and so may have had greater pupil disengagement, poorer teacher–pupil relations and higher substance use rates. Henderson et al. (2008) reported that schools in the East rather than the West of Scotland that focused primarily on academic attainment rather than caring and inclusivity tended to have the highest rates of teenage smoking. The quantitative measurement of value-added education differs from the qualitative assessment of an academic focus used by Henderson et al. (2008). The value-added measure not only includes an assessment of school-level truancy but is also an adjusted measure that takes into account the socio-demographic characteristics of each school's pupil population. Thus, schools with high value-added education scores occurred through the range of examination results and truancy rates and some high value-added schools would have had relatively low unadjusted academic attainment scores and relatively high unadjusted truancy rates. Previous studies (Aveyard et al., 2004b) and this study found that raw unadjusted school-level academic and truancy measures were unrelated to teenage substance use (data available on request).

The contradictory findings regarding the associations between value-added education and teenage substance use need to be resolved. However, if value-added education in Scotland is truly unrelated to pupils' perceptions of school and yet causally related to teenage substance use we could make two speculative proposals. First, Scottish schools may influence pupils' substance use through at least two pathways. One pathway focuses on school processes which influence traditional educational outcomes (examination success and truancy). The other focuses on school processes which positively influence pupils' school experience and school ethos. Both the investigation reported here and the investigation of Henderson et al. (2008) support the proposal of Gordon and Turner (2003) that the goals of traditional education and HPS agendas may be incompatible in Scotland and elsewhere. Second, we could speculate that the focus of the new Scottish Curriculum for Excellence on enabling a young person to realise four capacities (to be a successful learner, a confident individual, a responsible citizen and an effective contributor) may potentially positively influence teenage substance use.

### Substance use outcomes

The substance use outcomes used in this study replicated those used previously by West et al. (2004) and thereby facilitated comparisons between studies. The prevalence of drinking, drug use and smoking is however relatively high among UK teenagers (UNICEF, 2007). Conceptualising substance use outcomes among teenagers in the context of potentially more widespread substance use has proved challenging particularly when distinguishing harmful substance use from other forms of substance use (Perra, Fletcher, Bonell, Higgins, & McCrystal, 2011).

Henderson et al. (2008) proposed that substance use is a potential resource that teenagers may draw upon when constructing their identities. Fletcher et al. (2009) obtained evidence to support and extend this view. Thus, young people may draw on substance use when constructing identities at both the individual and group levels particularly when groups are hostile towards school (Fletcher et al., 2009). It is likely the converse is also true. Thus, teenagers may draw on abstention from substance use when actively constructing identities at both the individual and group levels particularly when they are empathetic and sympathetic towards school and schooling.

As proposed above, students in this study may have drawn on substance use as a resource in their rebellion or hostility towards the school ethos in high value-added schools in the West of Scotland. If this suggestion is valid then, drawing on the dose–response criteria of Bradford Hill (1965), we might speculate that there would be a positive relationship between value-added education and the perceived deviancy of the substance use outcome. If deviancy is defined via the numbers engaging in a particular behaviour, then monthly drinking in S4 was least deviant. Value-added education had the weakest association with this behaviour. Smoking is commonly perceived to be less deviant than illicit drug use but value-added education had a stronger relationship with current smoking than with ever use of illicit drugs. However, study participants needed to smoke on a regular or occasional basis to be categorised as smokers, but only needed to use drugs such as cannabis once to be categorised as illicit drug users. More participants were categorised as drug users than smokers, so, *based on these definitions*, *which are related to prevalence*, drug use could be considered relatively less deviant. Thus, our suggestion that high value-added education in this study might be more strongly associated with the most deviant behaviour is to some degree supported.

The proposal that within the context of the West of Scotland, there may be a positive relationship between value-added education and the perceived deviancy of the substance use is then, a tentative proposal that requires further consideration. However, this proposal is consistent with the empirical observations of Perra et al. (2011), who found that various aspects of school ethos had the strongest associations with the more harmful aspects of substance use.

### Disengagement and poor teacher–pupil relations

This discussion has focused on value-added education, but there are important findings related to other markers of school ethos. In all fully adjusted models, both disengagement and poorer teacher–pupil relationships remained significant moderate to strong risk factors for substance use. These effects were relatively large in comparison with the effects of other established teenage substance use risk factors. Additionally, other studies using different pupil engagement measures (Aveyard, Markham, Almond, Lancashire, & Cheng, 2003), found that independently of pupils' social backgrounds, schools that foster educational engagement have lower substance use. It is therefore important to understand what aspects of school organisation or culture promote these positive feelings that may, in turn, influence substance use. A potentially fruitful starting point could be the theoretical insights offered by Markham and Aveyard (2003) on how the school organisation, curriculum and pedagogic practice may be modified in order to promote positive feelings towards school. These theoretical insights resonate with the empirical findings of the Gatehouse project in Australia (Bond et al., 2004), the ‘Aban Aya’ project in the USA (Flay, Graumlich, Segawa, Burns, & Holliday, 2004) and Bonell et al. (2010) in England. However, as highlighted by Bonell et al. (2010), further investigations are required in order to gain deeper understanding of the underlying mechanisms and how these mechanisms relate to student substance use through positive and negative feelings towards school.

### Conclusion

Contrary to expectation and previous findings, schools providing higher value-added education were associated with a higher risk of substance use. Furthermore, we found that value-added education had no important relationship with our measures of pupils' perceptions of school, especially those related to the educational process. Five studies have found an association between substance use and value-added education. In four, the association was negative and in one study the association was positive. This pattern emphasises the importance of understanding the basis of the associations, which are likely to be best assessed via direct observations of educational management and its influences on pupils' perceptions. These assessments could potentially be usefully incorporated within future investigations that employ a similar design to the ‘*West of Scotland 11-16 Study*’, include a range of substance use measures, and are simultaneously conducted in both English and Scottish locations. The substance use measures would include more harmful measures such as binge drinking, public drunkenness and regular drunkenness as well as potentially less harmful substance use measures such as monthly drinking. Additionally, the analyses of the data would be extended to include sub-group analyses that focussed on detached and committed students. The proposed investigations could potentially provide more up-to-date data that inform important public health questions regarding the influence of school ethos on teenage substance use. They may also provide insights regarding the potentially variable influence of value-added education on teenage substance use.

## Sources of funding

The Medical Research Council funded the original cohort study but the subsequent analysis of the resulting data received no special funding. Paul Aveyard is part funded by the National Institute of Health Research and the UK Centre of Tobacco Control Studies. The UK Centre for Tobacco Control Studies is a UKCRC Public Health Research: Centre of Excellence. Funding from British Heart Foundation, Cancer Research UK, Economic and Social Research Council, Medical Research Council, and the Department of Health, under the auspices of the UK Clinical Research Collaboration is gratefully acknowledged. At the time these analyses were conducted, Robert Young, Helen Sweeting and Patrick West were funded by the UK Medical Research Council as part of the Youth and Health Programme at the Social and Public Health Sciences Unit. The funders had no input into the study design, collection, analysis, and interpretation of data, writing the report or in the decision to submit the paper.

## Figures and Tables

**Table 1:** Prevalence and school-level variance in Null and Individual Adjusted models of current smoking, monthly drinking and ever use of illicit drugs.

	Smoking		Drinking		Drugs	
	S2	S4	S2	S4	S2	S4
Prevalence (%)	10.6	25.3	31.0	62.5	17.5	40.3
Prevalence range (min-max %)	0.0–30.3	0.0–50.0	5.0–68.1	32.6–84.0	6.4–42.6	16.7–79.2
School-level variance						
Null Model	0.080	0.030	0.047	0.035	0.049	0.204
Individual Adjusted model	0.086	0	0.019	0.028	0.055	0.016

**Table 2:** The relationships between value-added education and pupils' perceptions of school life.^a^

	Coefficients	−95%CI	+95% CI	*p*
**S2**				
Poorer environment	−0.084	−0.174	0.006	0.065
Lower involvement*	−0.075	−0.142	−0.008	0.025*
Greater disengagement	−0.038	−0.105	0.029	0.201
Poorer teacher–pupil relations	0.0	−0.059	0.059	1
**S4**				
Poorer environment	0.078	−0.200	0.044	0.212
Lower involvement	−0.08	−0.164	0.004	0.063
Greater disengagement	−0.031	−0.092	−0.030	0.315
Poorer teacher–pupil relations	−0.035	−0.102	0.032	0.303

*p = <0.05.

a Changes in the standardised mediator scores for a 1 SD change in value-added in an adjusted multilevel regression model.

**Table 3:** The influence on substance use of value-added education and pupils' perceptions of school life.^a^

	Smoking		Drinking		Drugs	
	S2	S4	S2	S4	S2	S4
**Individual Adjusted model**						
School-level variance	0.086	0	0.019	0.028	0.055	0.016
**Model VA**						
School-level variance	0.070	0	0.015	0.028	0.052	0.016
**Value-added**						
OR (CI 95%)	1.28 (1.02–1.61)	1.13 (1.00–1.27)	1.11 (0.97–1.28)	0.94 (0.82–1.09)	1.14 (0.93–1.39)	1.02 (0.90–1.17)
*p*	0.034	0.049	0.135	0.403	0.197	0.717
**Model VA** **+** **PP**						
School-level variance	0.052	0	0.008	0.021	0.052	0.020
**Value-added**						
OR (CI 95%)	1.36 (1.08–1.70)	1.20 (1.05–1.36)	1.14 (0.99–1.30)	0.96 (0.84–1.11)	1.20 (0.98–1.47)	1.06 (0.91–1.20)
*p*	0.008	0.006	0.065	0.601	0.085	0.542
**Poorer environment**						
OR (CI 95%)	1.11 (0.89–1.39)	1.09 (0.93–1.27)	1.25 (1.08–1.45)	1.08 (0.94–1.24)	1.12 (0.93–1.35)	1.01 (0.88–1.16)
*p*	0.342	0.291	0.003	0.287	0.233	0.909
**Lower Involvement**						
OR (CI 95%)	0.96 (0.77–1.20)	0.94 (0.78–1.13)	0.93 (0.80–1.08)	0.95 (0.80–1.12)	0.97 (0.80–1.17)	0.92 (0.78–1.09)
*p*	0.714	0.517	0.344	0.531	0.758	0.338
**Disengagement**						
OR (CI 95%)	1.77 (1.41–2.23)	1.82 (1.52–2.17)	1.58 (1.35–1.85)	1.46 (1.24–1.70)	1.90 (1.55–2.31)	1.58 (1.35–1.85)
*p*	<0.001	<0.001	<0.001	<0.001	<0.001	<0.001
**Poorer T/P relations**						
OR (CI 95%)	1.60 (1.30–1.96)	1.40 (1.20–1.65)	1.35 (1.16–1.57)	1.36 (1.17–1.59)	1.33 (1.11–1.60)	1.52 (1.31–1.77)
*p*	<0.001	<0.001	<0.001	<0.001	0.002	<0.001

a Odds Ratios (95% confidence intervals) for 1SD increase in value-added scores.

**Table 4:** The influence on substance use of value-added education and school-level measures.^a^

	Smoking		Drinking		Drugs	
	S2	S4	S2	S4	S2	S4
**Model VA**						
School-level variance	0.070	0	0.015	0.028	0.052	0.016
**Value-added**						
OR (CI 95%)	1.28 (1.02–1.61)	1.13 (1.00–1.27)	1.11 (0.97–1.28)	0.94 (0.82–1.09)	1.14 (0.93–1.39)	1.02 (0.90–1.17)
*p*	0.034	0.049	0.135	0.403	0.197	0.717
**Model VA** **+** **SLF**						
School-level variance	0.003	0	0.011	0.022	0.020	0.012
**Value-added**						
OR (CI 95%)	1.35 (1.13–1.63)	1.18 (1.04–1.34)	1.14 (0.99–1.31)	0.98 (0.85–1.13)	1.21 (1.01–1.45)	1.07 (0.93–1.22)
*p*	0.001	0.011	0.078	0.762	0.038	0.347
**Larger school roll**						
OR (CI 95%)	1.21 (1.04–1.41)	1.04 (0.95–1.15)	1.05 (0.94–1.17)	1.10 (0.99–1.23)	1.14 (0.97–1.31)	1.04 (0.94–1.14)
*p*	0.016	0.372	0.392	0.074	0.075	0.456
**Non-denominational School**						
OR (CI 95%)	1.35 (0.43–4.24)	1.10 (0.51–2.37)	1.09 (0.48–2.49)	1.77 (0.83–3.75)	2.28 (0.70–7.39)	1.21 (0.57–2.58)
*p*	0.616	0.808	0.833	0.138	0.171	0.587
**Poorer aggregated school-level school ethos**						
OR (CI 95%)	4.60 (1.57–13.45)	1.93 (1.06–3.50)	1.49 (0.67–3.32)	1.16 (0.58–2.32)	3.78 (1.33–10.7)	1.68 (0.90–3.14)
*p*	0.005	0.032	0.334	0.671	0.012	0.104

a Odds ratios [95% confidence intervals] for 1SD increase in value-added scores.

**Table 5:** The influence on substance use of value-added education, pupils' perceptions and school-level measures.^a^

	Smoking		Drinking		Drugs	
	S2	S4	S2	S4	S2	S4
**Model VA**						
School-level variance	0.070	0	0.015	0.028	0.052	0.016
**Value-added**						
OR (CI 95%)	1.28 (1.02–1.61)	1.13 (1.00–1.27)	1.11 (0.97–1.28)	0.94 (0.82–1.09)	1.14 (0.93–1.39)	1.02 (0.90–1.17)
*p*	0.034	0.049	0.135	0.403	0.197	0.717
**Full model**						
School-level variance	0.003	0	0.008	0.012	0.034	0.020
**Value-added**						
OR (CI 95%)	1.39 (1.13–1.70)	1.20 (1.05–1.37)	1.12 (0.97–1.29)	0.98 (0.85–1.12)	1.24 (1.01–1.51)	1.06 (0.92–1.24)
*p*	0.002	0.008	0.117	0.724	0.038	0.411
**Poorer environment**						
OR (CI 95%)	1.08 (0.87–1.35)	1.09 (0.93–1.28)	1.25 (1.08–1.45)	1.10 (0.96–1.27)	1.01 (0.91–1.33)	1.01 (0.87–1.16)
*p*	0.480	0.303	0.003	0.169	0.324	0.933
**Lower Involvement**						
OR (CI 95%)	0.95 (0.76–1.18)	0.94 (0.78–1.13)	0.93 (0.80–1.08)	0.95 (0.80–1.12)	0.97 (0.80–1.17)	0.92 (0.77–1.09)
*p*	0.636	0.500	0.356	0.533	0.733	0.319
**Disengagement**						
OR (CI 95%)	1.74 (1.39–2.18)	1.82 (1.52–2.17)	1.58 (1.35–1.85)	1.47 (1.26–1.72)	1.87 (1.53–2.28)	1.59 (1.36–1.86)
*p*	<0.001	<0.001	<0.001	<0.001	<0.001	<0.001
**Poorer T/P relations**						
OR (CI 95%)	1.60 (1.31–1.96)	1.40 (1.20–1.65)	1.35 (1.16–1.57)	1.36 (1.17–1.59)	1.34 (1.12–1.61)	1.52 (1.31–1.77)
*p*	<0.001	<0.001	<0.001	<0.001	0.002	<0.001
**Larger School roll**						
OR (CI 95%)	1.18 (1.00–1.40)	1.02 (0.92–1.13)	1.04 (0.93–1.16)	1.11 (1.00–1.23)	1.14 (0.97–1.33)	1.02 (0.91–1.13)
*p*	0.054	0.700	0.479	0.044	0.105	0.779
**Non-denominational school**						
OR (CI 95%)	1.24 (0.33–4.70)	1.01 (0.45–2.29)	0.98 (0.40–2.37)	1.77 (0.83–3.79)	2.46 (0.66–9.18)	1.35 (0.60–3.03)
*p*	0.754	0.975	0.956	0.145	0.182	0.468
**Poorer aggregated school-level school ethos**						
OR (CI 95%)	2.50 (0.75–8.39)	1.04 (0.53–2.05)	0.84 (0.36–1.93)	0.66 (0.34–1.31)	2.15 (0.66–6.96)	1.13 (0.54–2.36)
*p*	0.137	0.903	0.679	0.235	0.201	0.722

a Odds ratios [95% confidence intervals] for 1SD increase in value-added scores.
